# Prognostic value of metastin expression in human pancreatic cancer

**DOI:** 10.1186/1756-9966-28-9

**Published:** 2009-01-21

**Authors:** Kazuyuki Nagai, Ryuichiro Doi, Fumihiko Katagiri, Tatsuo Ito, Atsushi Kida, Masayuki Koizumi, Toshihiko Masui, Yoshiya Kawaguchi, Kenji Tomita, Shinya Oishi, Nobutaka Fujii, Shinji Uemoto

**Affiliations:** 1 Department of Hepato-Biliary-Pancreatic Surgery and Transplantation, Kyoto University, Kyoto, Japan; 2 Department of Clinical Pharmacy, Oita University Hospital, Oita, Japan; 3 Graduate School of Pharmaceutical Sciences, Kyoto University, Kyoto, Japan

## Abstract

**Background:**

*KiSS-1 *was identified as a metastasis-suppressing gene in melanoma cells. The *KiSS-1 *gene product (metastin) was isolated from human placenta as the ligand of GPR54, a G-protein-coupled receptor. The role of metastin and GPR54 in tumor progression is not fully understood.

**Methods:**

We investigated the clinical significance of metastin and GPR54 expression in pancreatic cancer. We evaluated immunohistochemical expression of metastin and GPR54 in pancreatic ductal adenocarcinoma tissues obtained from 53 consecutive patients who underwent resection between July 2003 and May 2007 at Kyoto University Hospital. In 23 consecutive patients, the plasma metastin level was measured before surgery by enzyme immunoassay.

**Results:**

Strong immunohistochemical expression of metastin was detected in 13 tumors (24.5%), while strong expression of GPR54 was detected in 30 tumors (56.6%). Tumors that were negative for both metastin and GPR54 expression were significantly larger than tumors that were positive for either metastin or GPR54 (p = 0.047). Recurrence was less frequent in patients who had metastin-positive tumors compared with those who had metastin-negative tumors (38.5% versus 70.0%, p = 0.04). Strong expression of metastin and GPR54 was significantly correlated with longer survival (p = 0.02). Metastin expression by pancreatic cancer was an independent prognostic factor for longer survival (hazard ratio, 2.1; 95% confidence interval, 1.1–4.7; p = 0.03), and the patients with a high plasma metastin level (n = 6) did not die after surgical resection.

**Conclusion:**

Strong expression of metastin and GPR54 by pancreatic cancer is associated with longer survival. Metastin expression is an independent prognostic factor for the survival of pancreatic cancer patients. The plasma metastin level could become a noninvasive prognostic factor for the assessment of pancreatic cancer.

## Background

Pancreatic cancer remains a lethal disease and is the fourth to fifth leading cause of cancer-related death in the Western world, despite a significant reduction of the postoperative morbidity and mortality associated with pancreatectomy[1,2]. While surgical resection represents the only definitive option for cure of this disease and complete tumor resection is associated with longer survival, only 10% to 15% of patients have resectable disease[3,4]. Most patients with pancreatic cancer have locally advanced tumors, metastases, or both at the time of diagnosis. In addition, tumors frequently recur, even after margin-free curative resection, and most patients with recurrence have metastasis, which is often fatal. To improve the survival of patients with pancreatic cancer, we need a new strategy for the treatment of advanced disease that is unsuitable for surgical resection.

Metastasis is a multistep process in which tumor cells migrate through the stroma and invade a vessel, after which the cells are transported through the circulation to re-invade and proliferate at a distant site. Dozens of regulators influence each step of the metastatic cascade[5,6]. In 1996, *KiSS-1 *was identified as a human metastasis-suppressing gene in melanoma cells[7] and breast cancer cells[8]. Then, the *KiSS-1 *gene product was isolated from human placenta as the endogenous ligand of an orphan G-protein-coupled receptor known as GPR54[9], AXOR12[10], or hOT7T175[11]. *KiSS-1 *encodes a 145-amino acid peptide which is further processed to a C-terminally amidated peptide with 54 amino acids called metastin[11] or kisspeptin-54, as well as to peptides with 14 amino acids (kisspeptin-14) and 13 amino acids (kisspeptin-13)[9].

The bioactive sequence of the *KiSS-1 *gene product is the C-terminal 10 amino acids, metastin (45–54) (metastin-10 or kisspeptin-10)[12]. Metastin was shown to inhibit the chemotaxis and invasion of *GPR54*-transfected Chinese hamster ovary cells *in vitro*, while it inhibited the pulmonary metastasis of *GPR54*-transfected melanoma cells *in vivo*[11]. The prognostic relevance of *KiSS-1 *has been demonstrated for some solid tumors [13-21].

In addition to the inhibition of tumor metastasis, *KiSS-1 *shows neuroendocrine activity and has a role in the gonadotropin-releasing hormone cascade, puberty, placentation, and reproduction, as shown by recent studies[22,23]. In normal tissues, the highest level of *KiSS-1 *mRNA expression has been detected in the placenta, with moderate to weak expression in the central nervous system, testis, liver, pancreas, and intestine[7,10,11]. In the case of *GPR54 *mRNA, high levels of expression are found in the placenta, pancreas, and central nervous system [9-11].

We previously found that expression of *KiSS-1 *mRNA was lower and expression of *GPR54 *mRNA was higher in pancreatic cancer tissue compared with normal pancreatic tissue[24]. However, the clinical significance of *KiSS-1 *and *GPR54 *expression by pancreatic cancer remains unclear. We hypothesized high levels of *KiSS-1 *and *GPR54 *expression could be associated with better survival of pancreatic cancer patients. Therefore, we investigated immunohistochemical expression of the *KiSS-1 *gene product (metastin) and that of GPR54 in pancreatic cancer tissues obtained by surgical resection. We also measured plasma metastin levels in pancreatic cancer patients by using an enzyme immunoassay (EIA) that we previously established[25] and evaluated the clinical applicability of these two parameters.

## Methods

### Patients

A total of 53 consecutive patients with pancreatic cancer who underwent surgical resection between July 2003 and May 2007 at Kyoto University Hospital were studied. The diagnosis of ductal adenocarcinoma of the pancreas was confirmed histologically by at least two pathologists who examined the resected specimens. None of the patients received preoperative chemotherapy or radiation therapy, and all patients gave written informed consent to participation in the study. Follow-up information was obtained from the medical records or by direct contact with patients or their referring physicians.

We evaluated the following clinicopathological characteristics according to the sixth edition of the TNM classification of the international union against cancer (UICC)[26]: tumor location, tumor size, tumor extent (pT), lymph node metastasis (pN), pStage, histopathological grade (G), lymphatic invasion, venous invasion, perineural invasion, and residual tumor (R).

### Immunohistochemical staining for metastin and GPR54

Immunohistochemical staining of resected pancreatic tissues was done in 53 patients with ductal adenocarcinoma of the pancreas. We chose sections that contained cancer tissue and adjacent non-cancerous tissue in the same section.

**Figure 1:**
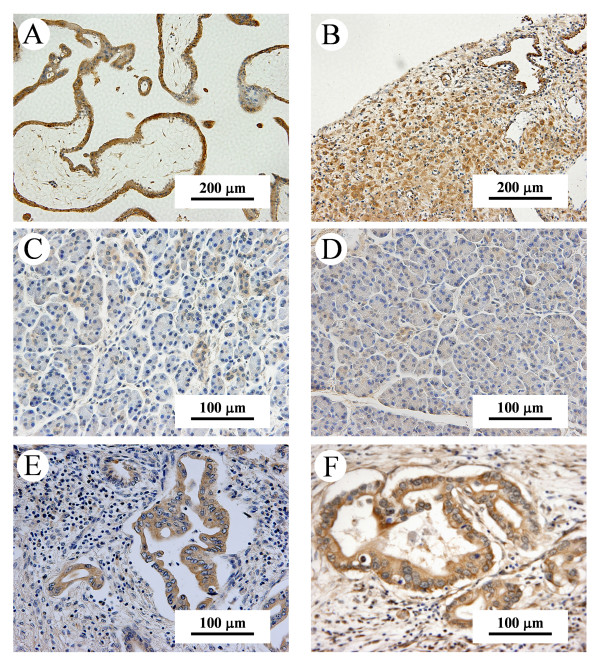
**Immunohistochemical staining of non-cancerous pancreatic tissues and pancreatic cancer tissues**. (A, B); Immunohistochemical staining of human placental tissues as a positive control. Tissues were stained with anti-metastin (A) and anti-GPR54 antibody (B). (Original magnification, × 200). (C, D); Non-cancerous and cancerous tissues were stained with anti-metastin and anti-GPR54 antibody. (Original magnification, × 400). Weak positivity of non-cancerous ductal cells for metastin (C) and GPR54 (D). (E, F); Pancreatic cancer tissues were stained with anti-metastin and anti-GPR54 antibody. Heterogeneous strong positive immunostaining of carcinoma cells for metastin (E) and GPR54 (F) are shown.

Paraffin-embedded tissue blocks were cut into 4 μm sections, dried overnight at 37°C, and then deparaffinized with xylene and rehydrated in a graded ethanol series. Sections were treated with Dako target retrieval solution (Dako, Carpinteria, CA, USA) before antigen retrieval was done by heating at 95°C for 40 min. Then the sections were cooled to room temperature, and were treated with dilute hydrogen peroxide to block endogenous peroxidase activity. Nonspecific binding was minimized by incubation with Dako protein block (Dako) for 30 min. Rabbit anti-human polyclonal antibodies for metastin (1–54)-Amide (catalogue number: H-048-59, Phoenix Pharmaceuticals, Inc., Burlingame, CA, USA) and GPR54 (375–398) (catalogue number: H-048-61, Phoenix Pharmaceuticals) were applied overnight at 4°C at a dilution of 1:400. On the next day, sections were incubated for 1 hr at room temperature with anti-rabbit IgG conjugated to a horseradish peroxidase (HRP) -labelled polymer (Dako Envision™ + System, Dako), treated with 3,3'-diaminobenzidine tetrahydrochloride (DAB), and counterstained with Mayer's hematoxylin. As a positive control, human placental tissue was stained with the anti-metastin and anti-GPR54 antibodies (Figure 1A, 1B). For negative control slides, the primary antibody was substituted with irrelevant rabbit serum.

### Assessment of metastin and GPR54 expression

Five fields (at a × 400 magnification) were randomly chosen to evaluate staining. The intensity of staining in cancer tissues was graded according to a 3-point scale as follows: 0 was weak; 1 was mild (the same staining intensity as that of non-cancerous pancreatic ducts as an internal control on each slide); and 2 was strong. The percentage of tumor cells showing each staining intensity was estimated to calculate an intensity score ([0 × %weak] + [1 × %mild] + [2 × %strong]) that could range from 0 to 200. A score ≥ 100 was defined as positive staining and a score <100 was defined as negative staining.

Then we compared clinicopathological characteristics between patients with positive and negative staining for metastin and GPR54.

### Blood sampling and EIA for plasma metastin

Plasma levels of metastin were measured by EIA, as described previously[25], in 23 consecutive patients who underwent resection between July 2006 and May 2007.

A blood sample was collected in the morning before surgery, placed in a chilled tube containing aprotinin (500 KIU/ml) and EDTA (1.2 mg/ml), and immediately centrifuged. The plasma thus obtained was diluted five-fold with 4% acetic acid (pH 4.0), and loaded onto a column with a C18 reversed-phase cartridge (Sep-Pak C18, Millipore, Milford, MA, USA). After washing with 4% acetic acid, peptides were eluted with 70% acetonitrile in 0.5% acetic acid (pH 4.0). The eluted samples were concentrated by spin-vacuum evaporation, lyophilized, and stored at -40°C until assay.

EIA was performed by the delayed-addition method with separation of bound and free antigens on anti-rabbit IgG-coated immunoplates. Human metastin (45–54) was conjugated with β-D-galactosidase using *N*-(ε-maleimidocaproyloxy)-succinimide, as reported previously[27]. The EIA was sensitive and specific for all bioactive *KiSS-1 *gene products (metastin, kisspeptin-14, and kisspeptin-13)[25].

The third quartile value was set as a cut-off for the plasma metastin level. We evaluated the association between the plasma level of metastin and metastin immunoreactivity in resected pancreatic cancer tissues, and also the associations between plasma metastin and the clinicopathological characteristics of the patients.

### Statistical analysis

Continuous variables are presented as the mean ± standard deviation or as the median and range. Comparison of the groups was done with the Mann-Whitney U test, while categorical variables were compared by the χ^2 ^test. Correlations between metastin and GPR54 immunoreactivity were investigated by calculation of Pearson's correlation coefficient (r) values and scatter plots with a linear regression line were drawn. An r value of 0–0.19 was defined as a very weak correlation, while 0.2–0.39 was weak, 0.40–0.59 was moderate, 0.6–0.79 was strong, and 0.8–1 was very strong. Overall survival curves were drawn by the Kaplan-Meier method, and were compared by the log-rank test. Prognostic factors for survival were examined by univariate and multivariate analyses using Cox's proportional hazards model. For all analyses, p < 0.05 was considered to be statistically significant.

## Results

### Demographic and clinicopathological characteristics

There were 25 men (47.2%) and 28 women (52.8%) with a mean age at diagnosis of 65.6 years (median age: 68 years; range: 32 – 86 years). The tumor was located in the head of the pancreas in 38 patients (71.7%), while it was found in the distal pancreas in 15 patients (28.3%). Pancreatoduodenectomy was performed in 36 patients (67.9%), while distal pancreatectomy was performed in 13 patients (24.5%), and total pancreatectomy in 4 patients (7.5%). On histopathological examination, one patient (1.9%) had pStage IA disease, three patients (5.7%) had pStage IB, 16 patients (30.2%) had pStage IIA, 29 patients (54.7%) had pStage IIB, and four patients (7.5%) had pStage IV.

Twenty-nine patients received adjuvant chemotherapy, which consisted of S-1 (n= 18), gemcitabine (n = 8), 5-fluorouracil (n = 2), and tegafur-uracil (n = 1). This was excluded from statistical analysis because of variations in the duration and type of chemotherapy.

### Immunostaining for metastin and GPR54

Pancreatic cancer tissues showed heterogenous immunoreactivity for metastin and GPR54 (Figure 1). Acinar cells and islet cells did not exhibit any immunoreactivity, while metastin and GPR54 were both weak or mildly positive in the cytoplasm of normal pancreatic ductal cells.

**Figure 2:**
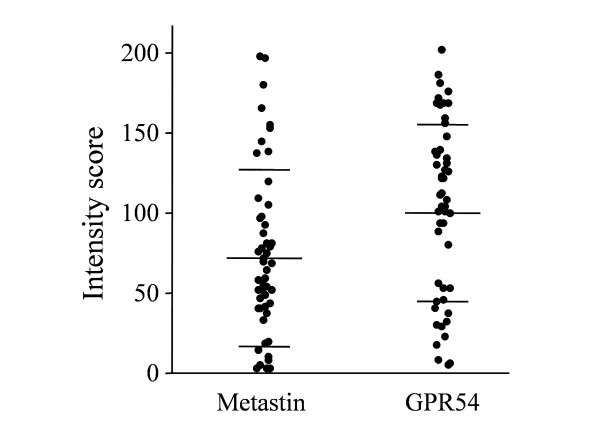
**Expression of metastin and GPR54 in pancreatic cancer tissues**. Immunoreactivity for metastin and GPR54 in resected pancreatic cancer tissues (n = 53) shown as the intensity score of each patient. The mean metastin intensity score was 72.1 ± 54.9 and that for GPR54 was 99.9 ± 55.1. The horizontal bar indicates the mean ± SD.

The mean intensity score for metastin was 72.1 ± 54.9 (n = 53) and that for GPR54 was 99.9 ± 55.1 (n = 53) (Figure 2).

**Figure 3:**
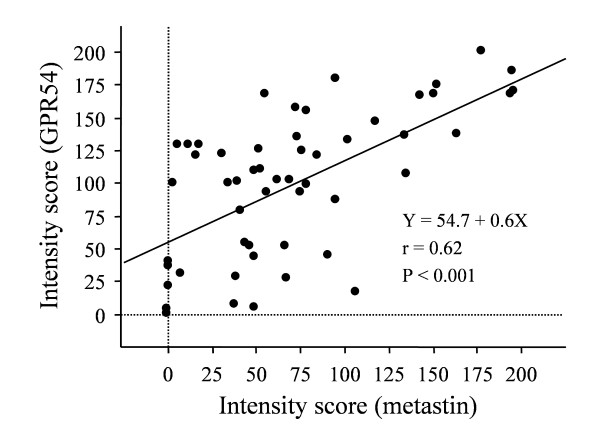
**Correlation between metastin and GPR54 expression in pancreatic cancer tissues**. Scatter plot showing the correlation between immunoreactivity for metastin and GPR54. A strong correlation was found (r = 0.62, p < 0.001).

Positive metastin staining was detected in 13 tumors (24.5%), while GPR54 was positive in 30 tumors (56.6%). Immunoreactivity for metastin and GPR54 showed a strong positive correlation (r = 0.62, p < 0.001; Fig. 3).

Demographic and clinicopathological characteristics showed no significant differences between patients whose tumors were positive or negative for metastin (Table 1), and the outcome was similar for GPR54 (Table 2). However, tumors that were negative for both metastin and GPR54 showed a significantly larger size than tumors positive for metastin and/or GPR54 (median of 2.5 cm and range of 0.8–5.0 cm versus median of 3.0 cm and range of 1.5–6.5 cm, p = 0.047).

**Table 1:** Comparison of the patients with pancreatic cancer who had positive immunostaining for metastin and those negative.

Characteristics	Positive for metastin	Negative for metastin	P value
	(n = 13)	(n = 40)	
Age	68.8 ± 7.2 (71, 56–78)	64.5 ± 10.5 (65.5, 32–86)	0.19
Gender			
Male	6	19	0.93
Female	7	21	
Location of tumor			
Pancreas head	8	30	0.35
Pancreas body-tail	5	10	
Size of tumor, cm	2.5 ± 0.9 (2.5, 1.2–4.5)	3.0 ± 1.2 (2.8, 0.8–6.5)	0.34
Histopathological grading			
G1	5	9	0.26
G2-4	8	31	
pT			
pT1, pT2	2	6	0.97
pT3	11	34	
pN			
pN0	6	15	0.58
pN1	7	25	
Lymphatic invasion			
Positive	7	24	0.70
Negative	6	16	
Venous invasion			
Positive	7	23	0.82
Negative	6	17	
Perineural invasion			
Positive	6	22	0.58
Negative	7	18	
pStage			
I, II	13	36	0.24
IV	0	4	
Residual tumor			
R0	11	28	0.30
R1	2	12	

Median and range are shown in parentheses.

**Table 2:** Comparison of the patients with pancreatic cancer who had positive immunostaining for GPR54 and those negative.

Characteristics	Positive for GPR54	Negative for GPR54	P value
	(n = 30)	(n = 23)	
Age	66.1 ± 8.7 (65.5, 49–86)	64.9 ± 11.5 (68.0, 32–80)	0.99
Gender			
Male	12	13	0.23
Female	18	10	
Location of tumor			
Pancreas head	21	17	0.75
Pancreas body-tail	9	6	
Size of tumor, cm	2.7 ± 1.0 (2.5, 0.8–5.0)	3.1 ± 1.2 (3.0, 1.2–6.5)	0.13
Histolopathological grading			
G1	10	4	0.19
G2-4	20	19	
pT			
pT1, pT2	6	2	0.25
pT3	24	21	
pN			
pN0	13	8	0.53
pN1	17	15	
Lymphatic invasion			
Positive	18	13	0.80
Negative	12	10	
Venous invasion			
Positive	18	12	0.57
Negative	12	11	
Perineural invasion			
Positive	15	13	0.64
Negative	15	10	
pStage			
I, II	29	20	0.18
IV	1	3	
Residual tumor			
R0	24	15	0.23
R1	6	8	

Median and range are shown in parentheses.

### Recurrence and survival

The median postoperative follow-up period was 18.5 months (range: 2.6–59.2 months). There were no operative deaths in this series. During the follow-up period, 33 patients (62.3%) showed recurrence and 25 patients (47.2%) died of their cancer. Recurrence was detected in the liver (n = 15), local region (n = 9), peritoneum (n = 9), lymph nodes (n = 5), lungs (n = 1), and bone (n = 1), while it was at an unknown location in 1 patient (elevated tumor marker). No patient died of any other disease or cause.

The recurrence rate was significantly lower in the patients whose tumors were positive for metastin than in those with negative tumors (38.5% versus 70.0%, p = 0.04) (Table 3). There were no significant differences of the recurrence rate at each site between the patients with metastin-positive and -negative tumors (Table 3), and the same was found for GPR54 (Table 4).

**Figure 4:**
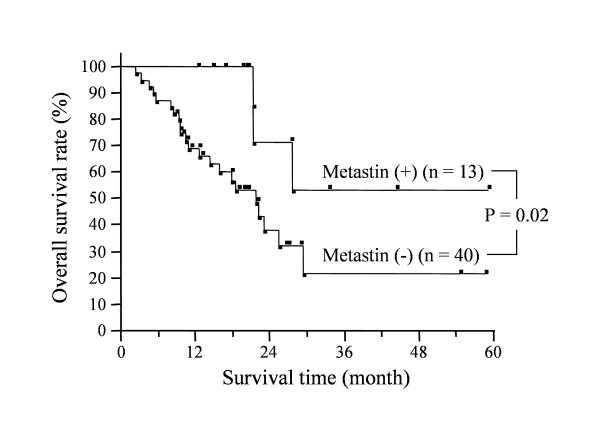
**Impact of metastin expression on survival time of pancreatic cancer patients**. Overall survival of patients whose tumors were positive (n = 13) or negative (n = 40) for metastin immunostaining. The survival of patients with positive tumors was significantly longer than that of patients with negative tumors (p = 0.02).

**Figure 5:**
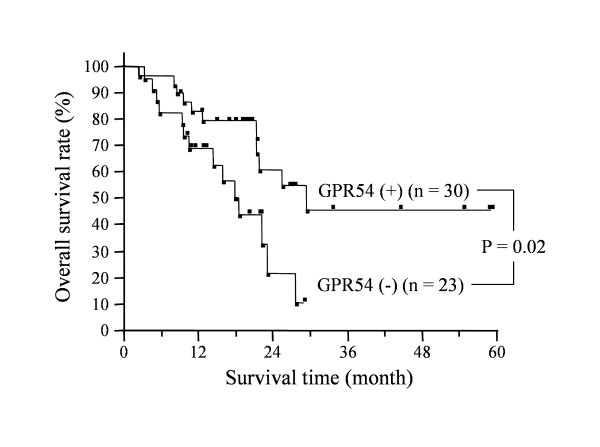
**Impact of GPR54 expression on survival time of pancreatic cancer patients**. Overall survival of patients whose tumors were positive (n = 30) or negative (n = 23) for GPR54 immunostaining. The survival of patients with tumors positive for GPR54 was significantly longer than that of those with negative tumors (p = 0.02).

The overall survival of patients whose tumors were positive for metastin was significantly longer than that of patients with negative tumors (p = 0.02) (Figure 4). Similarly, the overall survival of patients with tumors that were positive for GPR54 was significantly longer than that of patients with negative tumors (p = 0.02) (Figure 5).

**Table 3:** The rate and site of recurrence after resection of pancreatic cancer in relation to metastin expression.

	Metastin expression Positive (n = 13)	Metastin expression Negative (n = 40)	P value
Recurrence, n (%)	5 (38.5%)	28 (70.0%)	**0.04**
Site of recurrence			
Liver, n (%)	4 (30.8%)	11 (27.5%)	0.82
Local, n (%)	2 (15.4%)	7 (17.5%)	0.86
Peritoneum, n (%)	1 (7.7%)	8 (20.0%)	0.30
Lymph nodes, n (%)	1 (7.7%)	4 (10.0%)	0.80
Lungs, n (%)	0	1 (2.5%)	0.56
Bone, n (%)	0	1 (2.5%)	0.56
Unknown*, n (%)	0	1 (2.5%)	0.56

* Confirmed by elevated tumor marker during follow-up

**Table 4:** The rate and site of recurrence after resection of pancreatic cancer in relation to GPR54 expression.

	GPR54 expression Positive (n = 30)	GPR54 expression Negative (n = 23)	P value
Recurrence, n (%)	17 (56.7%)	16 (69.6%)	0.34
Site of recurrence			
Liver, n (%)	8 (26.7%)	7 (30.4%)	0.76
Local, n (%)	6 (20.0%)	3 (13.0%)	0.50
Peritoneum, n (%)	5 (16.7%)	4 (17.4%)	0.95
Lymph nodes, n (%)	2 (6.7%)	3 (13.0%)	0.43
Lungs, n (%)	1 (3.3%)	0	0.38
Bone, n (%)	0	1 (4.3%)	0.25
Unknown*, n (%)	0	1 (4.3%)	0.25

* Confirmed by elevated tumor marker during follow-up

### Prognostic factors according to multivariate analysis

Univariate and multivariate analysis were performed to identify parameters associated with overall survival according to the Cox proportional hazards model. The univariate analysis revealed the following five factors to be associated with survival: perineural invasion, pStage, residual tumor, metastin expression, and GPR54 expression. In the multivariate analysis, as well as the UICC pStage (I + II versus IV), overexpression of metastin was an independent prognostic factor for better survival (hazard ratio, 2.08; 95% confidence interval, 1.05–4.71; p = 0.03) (Table 5).

**Table 5:** Univariate and Multivariate analyses of factors associated with survival after resection in patients with pancreatic cancer.

	Univariate analysis		Multivariate analysis	
	
Characteristics	Hazard ratio (95% CI)	P value	Hazard ratio (95% CI)	P value
Age (continuous variables)	1.01 (0.97–1.1)	0.50	1.03 (0.97–1.1)	0.29
Gender (male versus female)	1.09 (0.73–1.6)	0.66	1.16 (0.73–1.9)	0.52
Location of tumor (head versus body-tail)	1.08 (0.72–1.7)	0.72	0.71 (0.40–1.3)	0.25
Size of tumor (continuous variables)	1.01 (0.97–1.0)	0.63	1.01 (0.96–1.1)	0.69
Histopathological grading (G1 versus G2-4)	1.05 (0.70–1.7)	0.80	0.92 (0.49–1.8)	0.79
pT (pT1, pT2 versus pT3)	1.62 (0.88–4.0)	0.14	2.07 (0.86–6.7)	0.11
pN (pN0 versus pN1)	1.27 (0.85–2.0)	0.25	1.01 (0.58–1.8)	0.97
Lymphatic invasion (positive versus negative)	1.20 (0.80–1.8)	0.33	0.97 (0.54–1.7)	0.92
Venous invasion (positive versus negative)	1.01 (0.68–1.5)	0.95	0.91 (0.52–1.6)	0.73
Perineural invasion (positive versus negative)	1.57 (1.1–2.4)	0.03	1.47 (0.85–2.7)	0.17
pStage (I, II versus IV)	3.16 (1.6–5.8)	0.002	2.70 (1.1–6.8)	0.03
Residual tumor (R0 versus R1)	1.61 (1.0–2.5)	0.03	1.60 (0.91–2.9)	0.10
Metastin expression (positive versus negative)	1.93 (1.1–4.0)	0.01	2.08 (1.1–4.7)	0.03
GPR54 expression (positive versus negative)	1.62 (1.1–2.5)	0.02	1.22 (0.74–2.0)	0.43

### Plasma metastin level

The mean plasma level of metastin before surgery was 22.7 ± 17.2 fmol/ml (median, 21.5 fmol/ml; range, 4.0–58.9 fmol/ml). Plasma metastin levels and the intensity score for metastin immunoreactivity in resected tissues showed a weak correlation (r = 0.23, p = 0.30). When we used the third quartile plasma metastin level (28.0 fmol/ml) as a cut-off value, there were no significant differences of demographics and clinicopathological characteristics between patients with a high (n = 6) or low (n = 17) plasma metastin level.

**Figure 6:**
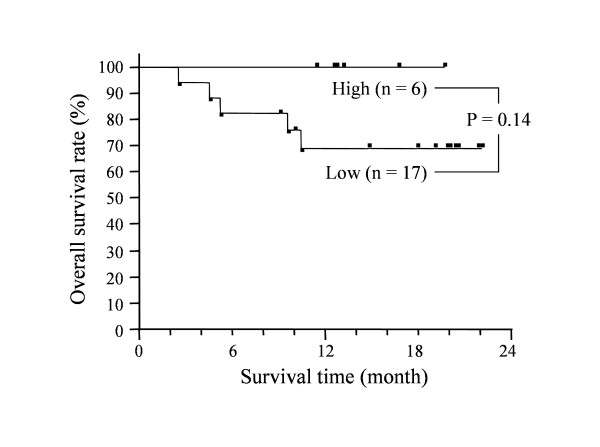
**Impact of plasma metastin levels on survival time of pancreatic cancer patients**. Overall survival of patients with high (n = 6) and low (n = 17) plasma metastin levels. There was no significant difference between the two groups (p = 0.14), but no patient with a high plasma metastin level died after surgery.

Overall survival curves of the patients with high and low plasma metastin levels are shown in Fig. 6. The median postoperative follow-up period was 14.8 months (range: 2.6–22.1 months, n = 23). While survival showed no significant difference between the two groups (p = 0.14), no patient with a high plasma metastin levels died after surgery (Figure 6).

## Discussion

In this study, we investigated the clinical significance of immunohistochemical metastin and GPR54 expression in resected pancreatic cancer tissues. We found that strong expression of metastin or GPR54 was associated with better survival, and metastin expression was an independent prognostic factor for longer survival of pancreatic cancer patients. Our results indicate that the metastin/GPR54 signaling system acts to suppress the growth of pancreatic cancer.

Recently, the prognostic relevance of *KiSS-1 *and *GPR54 *has been investigated in some solid tumors [13-21]. Most of these studies have shown that the *KiSS-1/GPR54 *system is negatively correlated with tumor progression. *KiSS-1 *has been demonstrated to act as a suppressor in melanoma[13], thyroid cancer[14], bladder cancer[16], gastric cancer[17], esophageal cancer[18], and ovarian cancer[20].

For example, Shirasaki et al[13] showed that downregulation of *KiSS-1 *is important for the progression of melanoma in vivo. Ringel et al[14] showed that *KiSS-1 *and *GPR54 *mRNA were overexpressed in papillary thyroid cancer compared with follicular cancer. In bladder cancer, loss of *KiSS-1 *expression is related to tumor progression[16]. In gastric cancer, lower expression of *KiSS-1 *mRNA is associated with venous invasion, distant metastasis, and tumor recurrence[17]. Furthermore, *KiSS-1 *is an independent prognostic marker for gastric cancer according to multivariate analysis [17]. Ikeguchi et al. [18] observed that loss of *KiSS-1 *mRNA, *GPR54 *mRNA, or both in esophageal squamous cell carcinoma was a significant predictor of lymph node metastasis. Finally, the survival of ovarian cancer patients with low *GPR54 *mRNA expression is significantly worse than that of those with high expression[20].

On the other hand, studies in patients with breast cancer[19] and hepatocellular carcinoma (HCC) [15,21] have yielded opposite results, with a positive association between increased KiSS-1 levels and disease progression. Martin et al. [19] found that *KiSS-1 *mRNA expression was increased in aggressive breast cancer. Ikeguchi et al. [15] reported that overexpression of *KiSS-1 *and *GPR54 *was correlated with the progression of HCC. Schmid et al. [21] performed an immunohistochemical study and concluded that high KiSS-1 expression was an independent prognostic factor for shorter survival of patients with HCC.

The mechanism by which the *KiSS-1/GPR54 *system regulates tumor progression still remains unclear, although various studies have revealed the downstream signaling pathways activated by *KiSS-1 *gene product. This might indicate that a complex signaling network exists with diverse physiological responses [23,28].

Stafford et al. [29] found that binding of *KiSS-1 *peptide to the receptor leads to activation of G-protein-activated phospholipase C, which suggested a direct relation of *KiSS-1 *to the Gαq-mediated phospholipase C-Ca^2+ ^signaling pathway. In addition, activation of GPR54 has been shown to cause an increase of intracellular calcium [9-11], arachidonic acid release [9], activation of mitogen-activated protein kinases (MAPKs), and activation of extracellular signal-regulated kinase (ERK) 1/2[9,14]. We have observed that exogenous metastin reduces migration of pancreatic cancer cells, while it induces the activation of ERK1 and p38[24]. Furthermore, the *KiSS-1 *product was shown to repress 92-kDa type 4 collagenase and matrix metalloproteinase (MMP)-9 expression by decreasing the binding of NF-κB to the promoter [30]. Bilban et al. [31] also found downregulation of MMP-2 activity by the *KiSS-1 *gene product in human trophoblasts, which implies an association between the tumor suppressor role of *KiSS-1 *suggested in this study and our previous report that activation of MMP-2 has a significant role in invasion and metastasis of pancreatic cancer[32].

*KiSS-1 *has also been shown to influence cell adhesion by forming focal adhesions through phosphorylation of focal adhesion kinase and paxillin [11], and an association between loss of KiSS-1 expression and E-cadherin expression was reported in bladder cancer [16].

In our series, there were no significant differences of clinicopathological characteristics between the patients whose tumors showed positive and negative metastin immunostaining, and the result was similar for GPR54. On the other hand, patients whose tumors showed negative immunoreactivity for both metastin and GPR54 had significantly larger tumors than those with lesions positive for either molecule. In addition, recurrence was more frequent in the patients with metastin-negative tumors than in those with metastin-positive tumors. These results suggest that pancreatic cancer loses metastin and GPR54 expression along with its progression. The *KiSS-1 *gene is mapped to chromosome 1q32-q41 [33] and *KiSS-1 *expression is regulated by genes located on chromosome 6 within the region 6q16.3-q23 [13,28]. These findings are consistent with the fact that loss of 6q, 8p, 9p, 12q, 17p, and 18q is frequently observed in pancreatic cancer[34,35].

Finally, we measured the plasma metastin level in 23 of our patients with pancreatic cancer. We previously found that the plasma metastin level of patients with pancreatic cancer is significantly higher than that of age- and gender-matched healthy volunteers (unpublished data), so we considered that there was potential to use plasma metastin as a novel tumor marker. In the present series, there was no significant difference of survival between the patients with high and low plasma metastin levels, but no patient with a high plasma metastin level died after surgery. Since the number of patients and the follow-up period are insufficient, more data and further investigation will be needed to clarify the value of measuring plasma metastin.

In this study, the plasma metastin level and metastin immunoreactivity in resected tumor tissues showed a weak correlation. It would be clinically useful if plasma metastin levels had prognostic significance because metastin expression in resected tumor tissues was shown to be a prognostic factor in this study.

## Conclusion

In conclusion, expression of metastin and GPR54 was associated with better survival of patients with pancreatic cancer. Metastin expression by cancer tissue was an independent prognostic factor for better survival. Furthermore, the serum metastin level could become a non-invasive prognostic tool for patients with pancreatic cancer.

## Competing interests

The authors declare that they have no competing interests.

## Authors' contributions

KN conceived of the study and performed immunohistochemical studies and measurements of serum metastin. RD conceived of the study, and participated in its design and coordination and helped to draft the manuscript. FK and TI conceived of the study and performed immunohistochemical studies. AK and MK conceived of the study and performed measurements of serum meatstin. TM, YK, KT, SO and NF conceived of the study and performed experiments on pancreatic cancer tissues. SU conceived of the study, and participated in its design.
